# Supplementary material for: Improving the Performance of Horseradish Peroxidase by Site-Directed Mutagenesis
Source: Int J Mol Sci. 2019 Feb 20;20(4):916. doi: 10.3390/ijms20040916 (PMC6412888; doi:10.3390/ijms20040916)
Supplement: Supplementary file 1 [file ijms-20-00916-s001.pdf]

Supplementary data

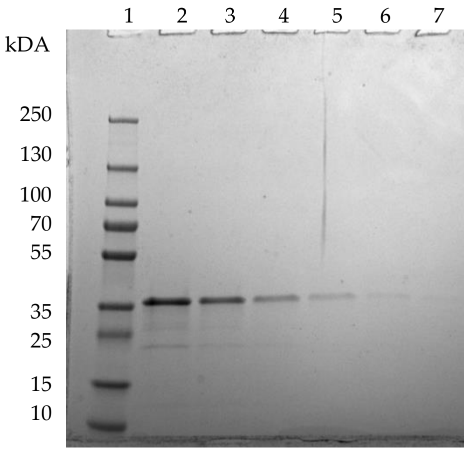

Supplementary Figure 1. rHRP dilution series used to quantify the amount of soluble rHRP in the IMAC eluate. The amount of soluble rHRP was calculated using the slope of the linear regression line of known rHRP concentrations plotted against the protein peak area. Lanes: 1, prestained protein ladder; 2, 3,500 ng; 3, 1,750 ng; 4, 875 ng; 5, 438 ng; 6, 220 ng; 7, 110 ng.
